# Cross-continental generalizability of EHR foundation model: predicting length of stay in an Italian hospital using US-trained embeddings

**DOI:** 10.3389/fdgth.2026.1956993

**Published:** 2026-10-09

**Authors:** Giovanna Nicora, Francesca Negri, Valentina Tibollo, Alberto Malovini, Arianna Dagliati, Lucia Sacchi, Federico Sottotetti, Paola Baiardi, Riccardo Bellazzi

**Affiliations:** 1 Department of Electrical, Computer and Biomedical Engineering, University of Pavia, Pavia, Italy; 2 IRCCS ICS Maugeri Hospital, Pavia, Italy

**Keywords:** distribution shift, external validation, generalist medical AI, length of stay prediction, transfer learning, transformer models

## Abstract

**Background:**

Clinical Foundations Models (FMs) hold the potential to exhibit generalist medical AI capabilities, and the ability to reduce the need for explicit feature engineering. However, their evaluation often relies on locally-limited datasets, raising questions regarding their generalizability across diverse global healthcare systems, populations, and clinical practices. This study assesses the cross-continental transferability of a US-trained FM, CLMBR-T-base, applied to a large, distinct, Italian hospital cohort to predict Length of Stay (LoS).

**Methods:**

We utilized a retrospective EHR dataset from IRCCS Maugeri, Pavia, Italy, comprising 99,884 admissions for 63,030 unique patients (2012–2025). To ensure interoperability, raw data were harmonized using the OMOP Common Data Model and converted to the Medical Event Data Standard (MEDS). Patient embeddings were generated zero-shot using the pretrained CLMBR-T-Base model. We benchmarked supervised regression models (Gradient Boosting, Multilayer Perceptron) trained on these embeddings against traditional count-based baselines to predict log-transformed LoS. We built different models trained on the complete hospital admissions set and stratified across four clinical macro-categories (Surgical, Rehabilitation, Oncology, Generic). Embedding cohesion was validated using latent space analysis (HDBSCAN clustering).

**Results:**

The best overall model utilizing FM embeddings achieved an R^2^ of 0.705. Performance varied by department type, proving lower in Rehabilitation (R^2^ = 0.36 for the best count-based model and R^2^ = 0.33 for the best embedding-based model) and Oncology (R^2^ = 0.17 for the best count-based model and R^2^ = 0.16 for the best embedding-based model). Latent space analysis confirmed that the FM embeddings preserved high semantic transferability, autonomously organizing Italian patients into clinically coherent groups.

**Conclusion:**

Clinical FMs trained on US data exhibit generalizability, effectively capturing clinical semantics across distinct international healthcare architectures when mediated by rigorous data standardization pipelines (OMOP/MEDS).

## 1 Introduction

In recent years, the rapid expansion of digital health infrastructure has made massive volumes of Electronic Health Record (EHR) data available for large-scale computation (1). This has fostered the development of clinical foundation models (FMs), which represent a move away from narrow artificial intelligence (AI) clinical applications toward versatile, adaptable AI systems (2–5) In fact, FMs are redefining how machine learning (ML) systems are developed and deployed across diverse domains (6, 7). Characterized by their self-supervised pretraining on massive, unlabelled datasets (8), FMs can be fine-tuned or adapted to solve a wide array of downstream tasks (9), moving the field away from narrow, task-specific algorithms (10). Applied to EHR, FMs offers unprecedented opportunities to capture the inherent complexity of patient clinical trajectories and integrate highly heterogeneous, multimodal data (11). These capabilities position EHR FMs as critical building blocks for the development of “Generalist Medical AI” (12) promising improved ML accuracy and enhanced robustness against temporal and distributional data shifts (13).

However, the translation of clinical FMs into real-world healthcare settings is hampered by substantial challenges related to generalizability, evaluation, and trust (14). An EHR-based foundation model inherently reflects the specific disease distributions, clinical guidelines, treatment pathways, and organizational structures of the healthcare system in which its training data was collected (15). Consequently, a critical, unresolved question remains: do models trained on specific patient populations generalize effectively to fundamentally different healthcare architectures? For instance, prominent models like CLMBR-T-Base (16, 17) have been trained on vast longitudinal datasets from US institutions such as Stanford Medicine, which operate within a private, insurance-based healthcare model. The same model has been validated on two cohorts from North America (5). Moreover, most clinical FMs have been tested on the same narrowly-scoped public datasets (18). Assessing whether these models can autonomously capture relevant clinical semantics and be transferred to diverse National Health Service is vital for understanding their global utility.

Furthermore, deploying these powerful tools across borders requires navigating complex and evolving regulatory landscapes, such as the European Union's AI Act, which demands rigorous standards for privacy, explainability, and safety. Interoperability serves as another fundamental bottleneck in evaluating and sharing these models (19). Bridging the gap between disparate healthcare ecosystems necessitates robust data standardization practices. Frameworks like the Observational Medical Outcomes Partnership (OMOP) Common Data Model (20), coupled with emerging standards like the Medical Event Data Standard (MEDS) (21), provide the critical infrastructure required to harmonize raw clinical data and facilitate cross-center model validation.

To address the urgent need for cross-country evaluation of clinical FMs, this study investigates the transferability of the US-trained CLMBR-T-Base model when applied to a distinct European cohort (Figure 1). We leverage a harmonized OMOP dataset comprising nearly 100,000 hospital admissions from the research hospital “IRCCS Istituti Clinici Scientifici Maugeri” in Pavia, Italy, to predict hospital Length of Stay (LoS).

**Figure 1:**
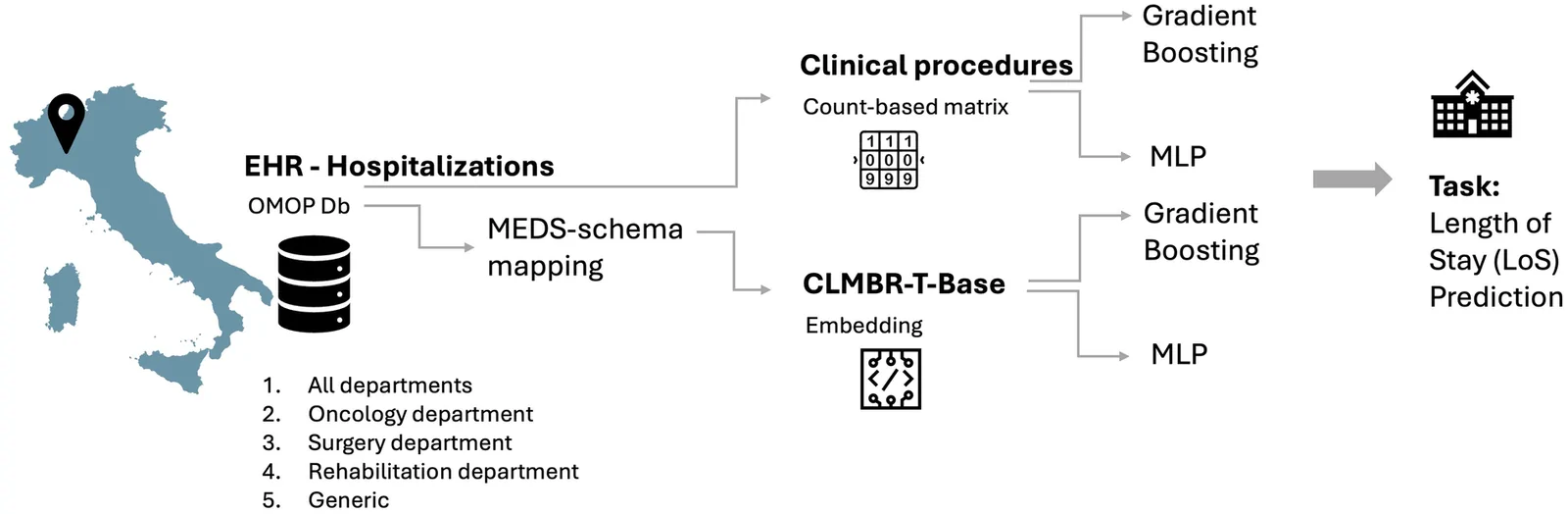
Workflow of the study. We considered hospitalizations from 2012 to 2025 reported in the OMOP database within the research hospital “IRCCS Istituti Clinici Scientifici Maugeri” in Pavia. Two data representation for each hospitalization were created: i) clinical procedures collected during the first day of hospitalization were used as features, and for each hospitalization the number of occurrence for each procedure were recorded; ii) the OMOP instance for first day of hospitalization were mapped into the MEDS schema, and the MEDS schema was provided as input to the pre-trained CLMBR-T-Base transformer model to extract the 768-size embedding vector. For patients with previous hospitalizations recorded at Maugeri, information from these previous admissions was also incorporated into both representations, following the vocabulary mapping described in the Methods. The two different representations were used to train two regression models (Gradient Boosting and a Multilayer perceptron (MLP)), to predict the length of stay.

Length of Stay (LoS) prediction is a vital task for optimizing hospital resource allocation and capacity planning. However, this task is inherently difficult; prior research consistently demonstrates that achieving high predictive performance is challenging, with reported R² values often limited to a 0.2 to 0.4 range, despite significant manual feature engineering (22, 23).

By benchmarking FM-derived embeddings against traditional count-based representations across diverse clinical departments, and by analyzing latent patient representations, this paper aims to provide empirical evidence on the cross-continental generalizability of clinical FMs on the critical task of predicting hospital length of stay, and underscore the vital role of global data standards in building trustworthy medical AI.

## 2 Materials and methods

### 2.1 Internal study population

To develop and validate the LoS predictive model, we utilized a clinical database originating from the research hospital IRCCS Istituti Clinici Scientifici Maugeri in Pavia, Italy. The dataset encompasses information on hospital admissions occurring between 2012 and 2025, distributed across 32 departments spanning diverse clinical and surgical specialties.

In total, the cohort includes 99,884 admissions corresponding to 63,030 unique patients. To ensure interoperability and standardization, the raw data—comprising diagnoses, procedures, pharmacological therapies, laboratory tests, clinical measurements, and demographic characteristics—were harmonized according to the semantic and structural conventions of the OMOP CDM.

To systematically analyze potential structural differences across highly heterogeneous clinical settings, the 32 original departments were subsequently aggregated into four macro-categories:

1. Surgical Departments: Settings where LoS is primarily dictated by surgical complexity and the potential onset of postoperative complications.
2. Rehabilitation Departments: Wards characterized by longer average stays and high variability, which are strongly linked to the patient's baseline functional status and recovery trajectory.
3. Oncology Departments: Units where the duration of stay is heavily influenced by specific therapeutic protocols (e.g., chemotherapy regimens) and individual responses to treatments.
4. Generic Departments: A heterogeneous grouping comprising units such as internal medicine, cardiology, and other specialties not encompassed by the aforementioned categories.

These macro-categories were defined to capture broad differences in the organizational and clinical context that may influence LoS, rather than to represent clinically homogeneous groups. The grouping was motivated by the specific characteristics of the study hospital and of the Italian healthcare setting. In particular, rehabilitation represents a major clinical focus of the hospital and is characterized by longer and more variable stays across heterogeneous patient populations. Oncology and surgical departments were retained as separate categories because their admissions follow distinct treatment pathways and organizational models, including the frequent use of day-hospital care for oncology and the predominance of scheduled procedures and postoperative management in surgery. The remaining specialties were grouped into the generic category because they were individually less represented in the cohort and did not provide sufficient sample size for separate analysis. This aggregation therefore provides a pragmatic balance between preserving clinically relevant differences in LoS patterns and obtaining sufficiently represented groups for comparative analysis.

### 2.2 Data preprocessing and CLMBRT-base embedding generation

To represent each hospitalization, we focus on the procedures and information collected during the first day of the hospitalization, which typically contains demographic data, diagnosis, and lab results. Specifically, the prediction cutoff was defined as the calendar date of admission. For each admission, information recorded up to midnight on the admission date was available to the model, together with the patient's clinical history within the Maugeri hospital, preceding the admission. To facilitate the use of the CLMBR-T-Base FM, we implemented a robust data processing pipeline to transform the relational data from the IRCCS Maugeri database into a ML-ready format.

A significant challenge in applying a model pre-trained on U.S. data to an Italian cohort is the discrepancy in clinical coding. The CLMBR-T-Base vocabulary is restricted to 26,249 tokens representing the most frequent codes at Stanford Medicine. In contrast, the Maugeri OMOP database contains hundreds of thousands of unique concepts. To bridge this gap, we implemented a recursive mapping procedure using the OMOP “concept_relationship” table. For codes not directly present in the model's vocabulary, we iteratively sought equivalents or more general parent concepts using relationships such as “is a”, “maps to”, and “concept same_as”. The overall mapping workflow used to align the Maugeri OMOP data with the CLMBR-T-Base vocabulary is summarized in Supplementary Figure S1. This process was carried out to a maximum depth of three levels to prevent excessive semantic generalization. This strategy achieved high coverage for critical domains (Supplementary Table S1), including 98.23% for diagnoses and 68.79% for laboratory measurements, ensuring that the model could “see” the majority of the Italian clinical events. Following vocabulary alignment, we converted the relational OMOP tables into the Medical Event Data Standard (MEDS) using the available ETL implementation (https://github.com/rvandewater/OMOP_MEDS). While traditional CDMs like OMOP are optimized for observational research, MEDS is an event-based schema specifically designed to facilitate large-scale ML on EHR. During this ETL process, we performed specific data cleaning to ensure compatibility with the MEDS schema. The MEDS representation included clinical information available in the OMOP database, including diagnoses and examinations/measurements recorded on the admission date, together with information from previous visits. Discharge planning and other administrative information related to the expected LoS were not included in the available clinical data. The final output consisted of Apache Arrow Parquet files, where each record contains a patient_id, static_measurements (e.g., birth and gender), and a chronologically ordered list of clinical events registered at the Maugeri research hospital.

To adapt these longitudinal records for the task of predicting hospital LoS, we restructured the patient-level MEDS into admission-level sequences. For each hospitalization, we defined a cutoff date corresponding to hospital admission day. To simulate the real-world clinical context where a prediction is made at admission, only events occurring on or before this cutoff were included. To assess whether model performance is influenced by the amount of longitudinal information available, we stratified performance according to the number of previous admissions per patient. This allowed us to evaluate whether admissions related to patientd with more extensive hospitalization histories, and therefore more available information, were systematically advantaged.

For patients with multiple hospitalizations, the MEDS sequence for each subsequent admission incorporated the available longitudinal clinical history recorded at Maugeri since 2012, together with the events recorded on the first day of the current admission. This history is limited to encounters captured in the Maugeri database and therefore does not include hospitalizations or clinical events occurring outside the institution. This admission-centric MEDS dataset provided the structured, sequential input required for CLMBR-T-Base to generate the dense, 768-dimensional embeddings used in our predictive models.

### 2.3 Machine learning for loS prediction

To evaluate the transferability of clinical representations, we modeled the hospital LoS prediction as a supervised regression task. The target variable was log-transformed prior to training to address the heavy right-skewness of the LoS distribution. Final predictions were back-transformed into the original temporal domain for clinical interpretation.

We compared two distinct patient representation paradigms:

1. Foundation Model Embeddings: Sequential clinical events were processed through the pre-trained CLMBR-T-Base transformer. We extracted the 768-dimensional dense embedding from the final row of the model's latent matrix, which represents the aggregated clinical state of the patient at the cutoff date.
2. Count-based Baseline: A high-dimensional, sparse matrix was constructed by calculating the frequency of occurrences for each clinical code within the first day of admission. To reduce dimensionality, feature selection was performed by removing codes present in more than 95% or fewer than 0.1% of admissions in the training set. For patients with multiple admissions, clinical codes from previous admissions were also included in the count-based representation, provided that the corresponding codes were retained during the OMOP-to-MEDS vocabulary mapping procedure. Thus, both the count-based and CLMBR-T-based representations incorporated available information from previous admissions, although this information was represented differently.

#### 2.3.1 ML architectures

For each representation, we implemented two supervised architectures: Gradient Boosting (GB) and a Multilayer Perceptron (MLP). The GB model utilized an additive ensemble of weak learners to capture non-linearities. The MLP consisted of fully connected layers using Rectified Linear Unit (ReLU) activations and was optimized via the Adam algorithm. To enhance robustness against outliers in the Italian cohort, the MLP was trained using Huber loss, which applies a quadratic penalty for small errors and a linear penalty for larger ones. As general baseline trained across all departments for LoS prediction, we also implemented a regression model using as covariates i) age, ii) gender, iii) department, iv) number of hospital admissions in the previous 6 months.

#### 2.3.2 Training and optimization

The dataset was partitioned into training (80%), validation (10%), and test (10%) sets. To prevent data leakage, we performed patient-level splitting, ensuring that all admissions from a single individual were assigned to the same subset. Hyperparameter optimization was conducted using Random Search over 10 independent iterations. See Supplementary Tables S2–S5 for the detailed selected hyperparameters. Performance for each configuration was estimated through 3-fold GroupKFold cross-validation, where the patient-id served as the grouping factor. LoS was log-transformed for model training and validation; R² was calculated on the log-transformed scale, while predictions were back-transformed to the original day scale for interpretation, and to compute the Median Absolute Error (MedAE), which represents the median absolute error, in days, between the predicted and observed LoS. All the analyses were performed using Python.

#### 2.3.3 Latent space evaluation

To verify if the US-trained FM autonomously organized the Italian patient data into clinically coherent groups, we applied HDBSCAN clustering directly to the high-dimensional embeddings. This density-based analysis was performed without prior dimensionality reduction to preserve the intrinsic geometric properties of the latent space. The resulting clusters were evaluated for intra-cluster cosine similarity and separation between centroids.

## 3 Results

### 3.1 Data preprocessing and exploratory analysis

The data preprocessing pipeline successfully harmonized and mapped the IRCCS Maugeri clinical data into a structured format compatible with the CLMBR-T-Base foundation model. The results of this process characterize the final study cohort and the semantic alignment between the Italian clinical practices and the US-centric model vocabulary.

#### 3.1.1 Cohort selection and filtering

The initial database comprised 99,884 admissions from 63,030 patients between 2012 and 2025. After applying the exclusion criteria—removing palliative care, and day hospital stays—and filtering for admissions lasting longer than 24 h to ensure a clear distinction between the informative cutoff and the predictive outcome, the total volume of admissions was reduced by 18.54%. Episodes shorter than 24 h were excluded because, in the Italian healthcare setting, these episodes correspond to planned day-hospital/day-service activities rather than inpatient hospitalizations with an overnight stay. They therefore represent a different type of care episode from the inpatient LoS outcome considered in this study. The final processed dataset for modeling included 81,371 admissions corresponding to 52,585 unique patients. On average, there were 1.55 admissions per patient, with a significant concentration of records in the rehabilitation (32,947) and general (31,452) departments (Table 1).

**Table 1:** General statistics about hospital admissions.

Departments	Admissions	Patients	Admissionsper patients (mean ± SD)	Admissionsper year (mean ± SD)	Mean ± SD admissions for department	LoS for department (mean ± SD, median[Q1-Q3]
Total	81,371	52,585	1.55 ± 1.32	5,812.2 ± 1,239.3	2,542.8 ± 3,281	17.8 ± 24.21 11 (4–22)
Surgery	9,232	8,413	1.1 ± 0.37	659.4 ± 140.5	3,077.3 ± 4,615.1	3.75 ± 4.2 2 (2–3)
Oncology	7,740	4,264	1.82 ± 1.83	552.9 ± 269.4	2,580.0 ± 3,800.2	9.29 ± 7.58 7 (4–12)
Rehabilitation	32,947	24,077	1.37 ± 1.04	2,353.4 ± 599.9	3,294.7 ± 3,574.1	29.95 ± 26.05 22 (16–35)
Generic	31,452	21,704	1.45 ± 1.11	2,246.6 ± 464.6	2,096.8 ± 3,062.4	11.28 ± 22.66 6 (3–12)

#### 3.1.2 Vocabulary alignment and coverage

A critical result of the preprocessing stage was the effectiveness of the recursive mapping strategy in bridging the gap between the Maugeri database's concepts and the model's 26,249-token vocabulary. The implementation of hierarchical and equivalence mapping (up to three levels deep) achieved substantial coverage across primary clinical domains.

The highest alignment was observed in diagnoses (Condition Occurrence), where 98.23% of the Italian clinical codes were successfully mapped to recognized tokens in the CLMBR-T-Base vocabulary. Procedures and Laboratory Measurements also showed robust alignment, with 74.75% and 68.79% coverage, respectively. However, the drug exposure domain presented the greatest challenge, with only 35.61% coverage, likely due to the high granularity of pharmaceutical lexicons and differences between European and U.S. medication standards. Following all filtering and mapping procedures, the final longitudinal patient records were represented using a refined set of 4,017 unique clinical tokens.

#### 3.1.3 Clinical distribution

The analysis of the mapped data revealed a high concentration of clinical information within a relatively small subset of codes. For instance, in the Condition Occurrence table, while there were 3,490 distinct concept IDs, just 55 codes accounted for 50% of all recorded diagnoses, and 890 codes covered 95%. Similar patterns were observed in measurements, where 21 codes explained 50% of the recorded lab results.

In the specialized Oncology department, this concentration was even more pronounced: the top 10 codes in the observation table accounted for 99.2% of all records. This indicates that while the overall dataset is broad, the trajectories of the Italian cohort are often defined by a core set of frequent clinical events.

Length of stay distribution also varies across departments (Supplementary Figures S1–S5), with rehabilitation showing the highest LoS.

#### 3.1.4 Exploratory characterization of the representation learned by the foundation model

We performed unsupervised analysis by applying the HDBSCAN clustering algorithm directly to the CLMBRT-Base 768-dimensional embeddings. The algorithm identified three distinct patient groups without prior dimensionality reduction (Figure 2).

**Figure 2:**
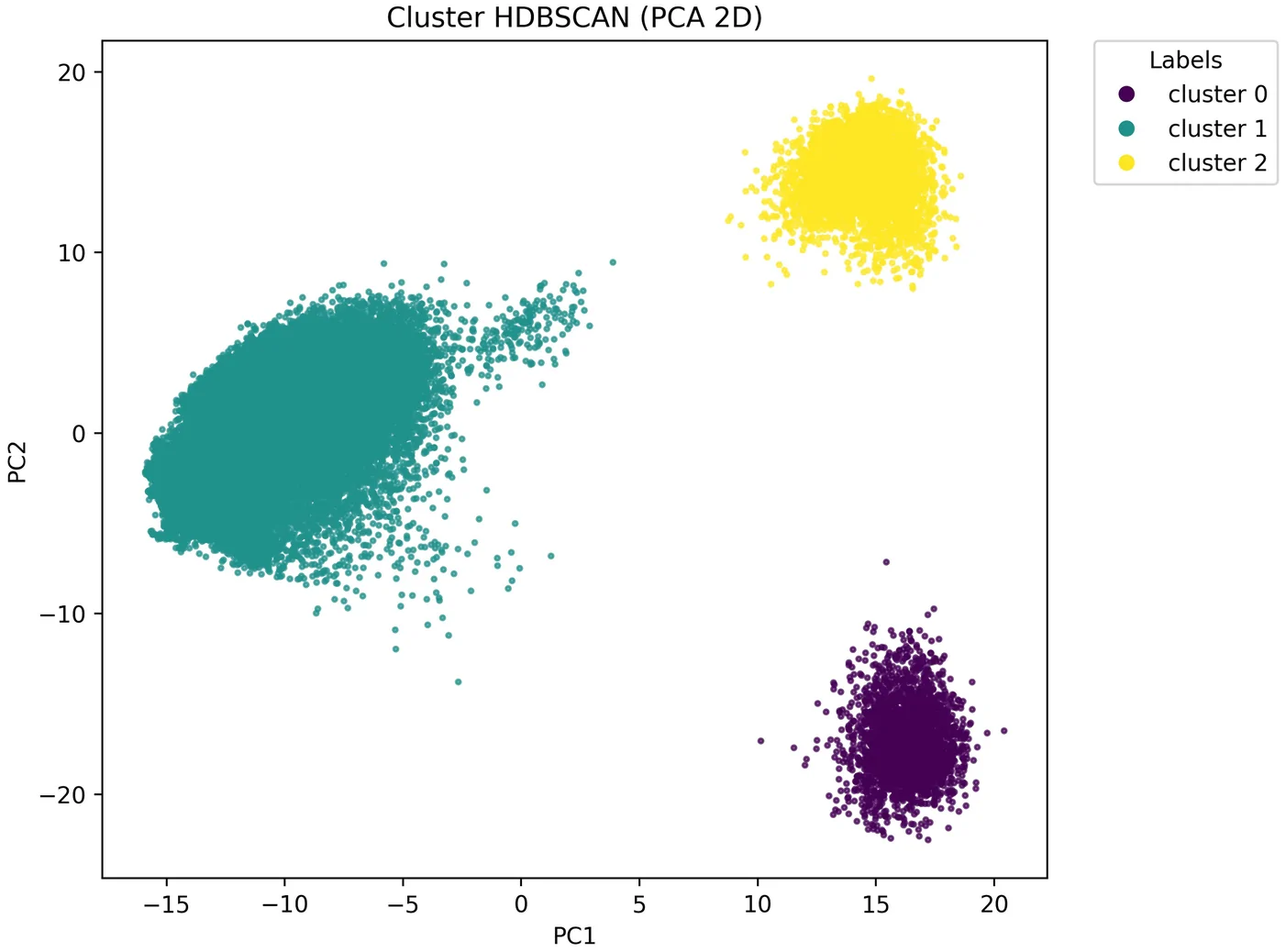
Clusters identified through unsupervised HDBSCAN using the embedded representation.

Cluster 1 (the largest group) showed the highest intra-cluster cosine similarity (0.83), indicating a high degree of clinical homogeneity in the representations. The clusters were well-separated, with a median LoS ranging from 7 days in Cluster 0 to 21 days in Cluster 2 (Table 2).

**Table 2:** Median LoS for each cluster, and their cosine similarity.

Statistics	Cluster 0	Cluster 1	Cluster 2
Median LoS	7	12	21
Cosine similarity (intra-cluster)	0.76	0.83	0.76

This natural organization of Italian patients into meaningful groups in the latent space provides evidence that the transferred representation captures structure associated with patient characteristics in the Italian cohort

### 3.2 Length of stay prediction

#### 3.2.1 Overall model performance

In the comprehensive model encompassing all hospital departments, the MLP trained on CLMBR-T-Base embeddings emerged as the most accurate configuration, achieving an R^2^ of 0.705 (Figure 2). On the overall dataset, this model demonstrated a slightly increased ability to capture complex clinical trajectories compared to the best count-based baseline (Gradient Boosting with matrix), which reached an R^2^ of 0.685. Department-specific R² values were lower, suggesting that part of the overall performance may reflect heterogeneity in LoS across clinical settings. The MLP-embedding pipeline also recorded the lowest error rates, with a Median Absolute Error (MedAE) of 3.0 days, while the MLP-count based model had 3.1 MedAE. The GB-count based had a MedAE of 3.2 days, while the embedding-based GB had a slightly higher MedAE (3.5 days). The error distribution showed a tendency for prediction errors to increase with observed LoS (Supplementary Figure S6). Predictions for shorter hospitalizations were generally more concentrated around the zero-error line, whereas longer hospitalizations were associated with larger and more dispersed errors, including a tendency toward overestimation at the highest LoS values. The baseline models with few covariates reached an R^2^ of 0.5 (Figure 3).

**Figure 3:**
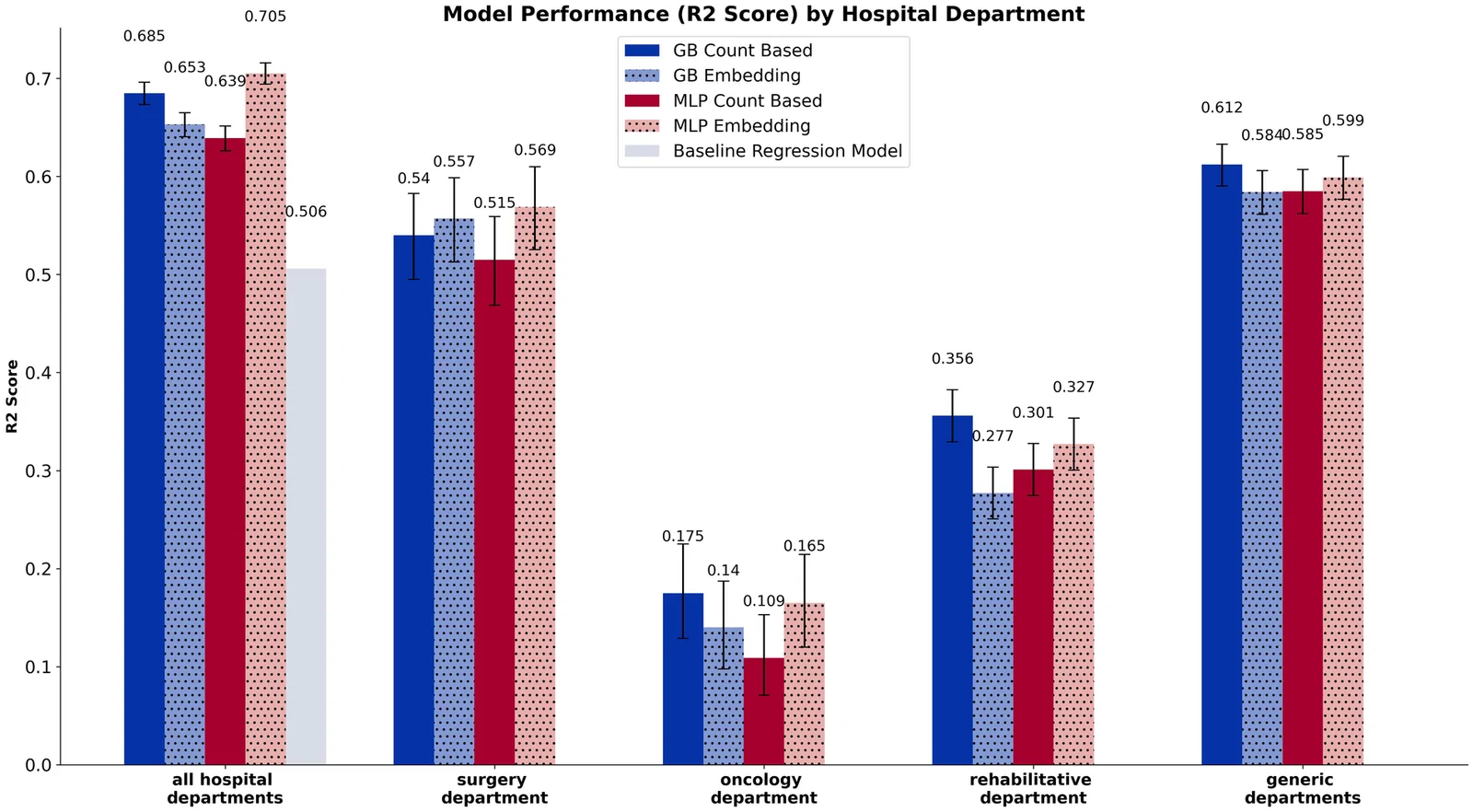
Models performance in terms of R^2^ (with 95% CI), to predict length of stays across various departments.

A temporal validity test confirmed the model's robustness; when trained on data prior to 2019 (specifically, 51,675 admissions) and tested on the post-2020 (COVID-19 era) cohort (with 29,696 admissions), the embedding-based approach maintained a R^2^ of 0.65. On the same temporal validity test, the best count-based counterpart achieved a R^2^ of 0.608.

#### 3.2.2 Department-specific results

The predictive performance varied significantly across clinical specialties, reflecting the differing nature of their care pathways.

In the surgery use case, both embedding-based models outperformed their matrix counterparts, with the MLP reaching an R^2^ of 0.569. The results indicate that the FM effectively encodes the standardized nature of surgical interventions. However, MedAE was low and similar across models, with a value of 0.4 days for all models except the MLP-embedding model, which achieved a MedAE of 0.3 days. In the General Medicine use case, performance remained strong and relatively stable across configurations, with the MLP-embedding model achieving an R^2^ of 0.599. MedAE was 1.7 days for the count-based models and 1.8 days for the embedding-based model. The Rehabilitation department presented higher complexity, where the GB model on count-based matrices actually performed best (R^2^ = 0.356). MedAE ranged from 6.3 days for the GB and MLP count-based models to 6.5 days for the MLP-embedding model and 6.8 days for the GB-embedding model. The higher variability in chronic care recovery times proved more challenging for the US-trained FM representations. The Oncology use case showed the lowest predictability across all models (R^2^ ranging from 0.109 to 0.175). Analysis suggests this is due to high information concentration—where 10 codes cover 99.2% of observations—and the absence of critical non-structured data like disease staging in the current OMOP mapping. The MLP-embedding model achieved the lowest MedAE (2.6 days), while the remaining models had a MedAE of 2.8 days.

Overall, these results highlight differences in both explained variance and absolute prediction error across departments. In particular, the relatively high R² observed in Surgery and General Medicine was accompanied by substantially lower MedAE than in Rehabilitation.

#### 3.2.3 Impact of admission history on model performance

Table 3 reports the models' performance (in terms of R^2^) stratified by patients who had only one admission and those that had multiple admissions. Interestingly, the performance is consistently higher for single-admission cases, both using embedding and count-based representations. Across all evaluated representations, model performance was higher for patients with a single admission than for those with multiple admissions. This pattern was consistent across the different representations, despite the greater amount of longitudinal information available for patients with multiple admissions. As for patients with multiple admissions, codes from previous admissions were also included in the count-based representation (provided that the corresponding codes are represented within the CLMBR-T-base vocabulary, including mapping), the observed difference in performance between the single- and multiple-admission strata should not be interpreted as evidence that the additional history available to the FM explains the performance difference. Rather, the results indicate that the relative performance of the two representations varies according to the patient's admission history. In particular, the lower performance of the embedding-based models in the multiple-admission subgroup suggests that the advantage observed in the overall cohort cannot simply be attributed to the availability of longitudinal history.

**Table 3:** R2 [95% CI performance] stratified according to patients’ admission history, comparing single-admission and multiple-admissions.

Representation Type	Model	Single Admission (*N* = 3,558)	Multiple Admissions (*N* = 4,543)
Count Based	GB	0.693 [0.68–0.71]	0.679 [0.66–0.69]
	MLP	0.726 [0.71–0.74]	0.556 [0.54–0.57]
Embedding	GB	0.765 [0.75–0.78]	0.546 [0.54–0.58]
	MLP	0.797 [0.78–0.81]	0.618 [0.6–0.63]

Number of single and multiple admissions are reported.

## 4 Discussion

This study demonstrates that a clinical foundation model (CLMBR-T-Base) pre-trained on U.S. patient data, effectively generalizes to an Italian healthcare context. The MLP-embedding pipeline achieved an R² of 0.705, which is within the range of state-of-the-art results reported for LoS prediction in previous studies (22, 23), while providing a modest improvement over the traditional count-based baseline (R^2^ = 0.685). This result suggests that the FM latent representations capture information relevant to LoS prediction, while reducing the need for explicit feature engineering and missing-value handling required by conventional count-based representations, provided that clinical data are collected and stored in a standardized way. While the count-based approach required missing clinical codes to be explicitly represented, CLMBR-T-Base operates directly on the available longitudinal clinical event sequences, avoiding the need to construct and impute a high-dimensional feature matrix. However, our findings indicate that the utility of pretrained clinical representation may depend on the clinical context and characteristics of the target population. In some department (such as oncology and rehabilitation), simple count-based representations achieved higher performance than the embedding-based approach. This may partly reflect differences between the data and clinical pathways represented during pretraining and those of the target healthcare setting. Further evaluation across institutions and healthcare systems, as well as domain-specific adaptation of pretrained representation, is needed to assess the generalizability of the results. The lower performance observed among patients with multiple admissions, consistently across representations, may suggest that a greater amount of longitudinal information does not necessarily provide additional predictive value for the length of stay (Table 3). This finding could reflect the greater clinical heterogeneity of patients with repeated admissions or the presence of historical information that is less informative for the current hospitalization. However, these explanations remain speculative and would require further investigation.

A key contribution of this work is the validation of data standardization as a bridge for cross-continental AI. The use of the OMOP Common Data Model and the MEDS schema was essential to harmonize disparate clinical lexicons, allowing a model trained in California to interpret data from Lombardy. Furthermore, the model's temporal robustness—maintaining an R^2^ of 0.65 when tested on the post-2020 cohort—indicates that FM-derived representations are resistant to major organizational shifts, such as those induced by the COVID-19 pandemic.

The unsupervised clustering analysis provides further evidence of the model's semantic depth. Without local fine-tuning, the FM organized Italian patients into three distinct clusters with significantly different median LoS (7, 12, and 21 days), suggesting that the latent space autonomously encodes clinical severity and recovery trajectories.

However, performance was not uniform across all departments. While surgery and general medicine showed high predictability, oncology remained a significant challenge (R^2^ ≅ 0.17). Our analysis suggests this failure stems from information concentration (where 10 codes cover over 99% of observations) and the absence of critical variables in structured EHR, such as cancer staging or treatment response, which are often relegated to unstructured clinical notes. Additionally, the low coverage of the drug vocabulary (35.6%) highlights the persistent challenge of reconciling different international pharmaceutical standards. Crucially, the MedAE analysis provides a more nuanced assessment of the practical value of the approach: while the results indicate potential for LoS prediction, the observed prediction errors remain too large to support clinical deployment at this stage. Further validation and improvement of prediction accuracy would therefore be required before considering its use in clinical practice. The exclusion of planned day-hospital/day-service episodes may limit the generalizability of the findings to inpatient admissions and should be considered when interpreting the results. Finally, this study was conducted in a single Italian research hospital with a specific distribution of clinical departments and patient characteristics. These institutional and cohort-specific features may influence both the observed length-of-stay patterns and model performance, limiting the generalizability of the findings to other Italian hospitals and healthcare systems with different organizational and clinical settings. The prediction time point also represents an important characteristic of the proposed setting. Predictions were generated using information available on the calendar day of admission, together with the patient's previous clinical history. Thus, the amount and type of information available at prediction may vary depending on the timing and circumstances of admission, and the model should be interpreted as an early-admission prediction approach that can provide information for early patient management and resource allocation. The aggregation of several heterogeneous specialties into the “Generic” category may mask department-specific differences in LoS patterns and should therefore be considered when interpreting the corresponding results. Moreover, FMs like CLMBR-T-Base still face significant challenges in terms of explainability, making it difficult to identify which variables or EHR data configurations are most important for their predictions.

## 5 Conclusion

Our results provide empirical evidence that Generalist Medical AI is a viable goal, provided it is supported by rigorous data standards. We show that pre-trained clinical FMs can be successfully deployed in new geographical and regulatory settings with minimal local infrastructure, potentially democratizing access to advanced predictive tools for hospitals with limited computational resources.

Future research should focus on local fine-tuning to bridge vocabulary gaps and the integration of multimodal data (e.g., clinical notes and imaging) to capture the nuances of specialized fields like oncology. Ultimately, integrating these robust representations into hospital information systems could significantly improve resource allocation and bed management, paving the way for a more predictive and personalized approach to inpatient care.

## Data Availability

The data analyzed in this study is subject to the following licenses/restrictions: Due to the sensitive nature of the hospital data used in this work, the raw datasets cannot be publicly shared to protect patient confidentiality and comply with privacy regulations. Requests to access these datasets should be directed to giovanna.nicora@unipv.it.
