# Supplementary material for: Cross-continental generalizability of EHR foundation model: predicting length of stay in an Italian hospital using US-trained embeddings
Source: Front Digit Health. 2026 Oct 9;8:1956993. doi: 10.3389/fdgth.2026.1956993 (PMC13690950; doi:10.3389/fdgth.2026.1956993)
Supplement: Supplementary file 1 [file Datasheet1.docx]

Supplementary Material

# 1 Supplementary Methods

| **Table** | **Coverage** |
| --- | --- |
| Condition_occurrence | 98.23% |
| observation | 52.48% |
| measurement | 68.79% |
| procedure | 74.75% |
| Drug_exposure | 35.61% |

# Supplementary Table S1: coverage of the Maugeri OMOP database within the CLMBRT-Base vocabulary across OMOP tables

| **Department** | **subsample** | **n_estim** | **min_leaf** | **max_feat** | **max_depth** | **lr** |
| --- | --- | --- | --- | --- | --- | --- |
| All hospital department | 1 | 300 | 20 | 1 | 10 | 0.05 |
| Surgery | 1 | 300 | 20 | 1 | 3 | 0.1 |
| Oncology | 0.8 | 100 | 20 | 1 | 5 | 0.05 |
| Rehabilitation | 0.8 | 100 | 20 | 1 | 10 | 0.05 |
| Generic | 0.8 | 100 | 20 | 1 | 10 | 0.05 |

# Supplementary Table S2: Best Hyperparameters for the Gradient Boosting trained on the count-based matrix

| **Department** | **subsample** | **n_estim** | **min_leaf** | **max_feat** | **max_depth** | **lr** |
| --- | --- | --- | --- | --- | --- | --- |
| All hospital department | 0.8 | 500 | 100 | sqrt | 10 | 0.01 |
| Surgery | 0.8 | 100 | 50 | 1 | 5 | 0.05 |
| Oncology | 0.8 | 200 | 50 | 1 | 3 | 0.05 |
| Rehabilitation | 0.8 | 300 | 100 | sqrt | 5 | 0.05 |
| Generic | 0.8 | 500 | 100 | sqrt | 10 | 0.05 |

# Supplementary Table S3: Best Hyperparameters for the Gradient Boosting trained on the CLMBR-T-base embeddings

| **Department** | **hidden dims** | **dropout** | **epochs** | **patience** | **lr** | **weight decay** |
| --- | --- | --- | --- | --- | --- | --- |
| All hospital department | 128-64-32 | 0.3 | 200 | 30 | 3.00E-04 | 3.00E-04 |
| Surgery | 64-32 | 0.3 | 200 | 30 | 3.00E-04 | 1.00E-02 |
| Oncology | 64-32 | 0.3 | 200 | 30 | 3.00E-03 | 1.00E-02 |
| Rehabilitation | 128-64 | 0.3 | 200 | 30 | 1.00E-04 | 1.00E-03 |
| Generic | 128-64 | 0.3 | 200 | 30 | 1.00E-04 | 1.00E-03 |

# Supplementary Table S4: Best Hyperparameters for the MultiLayer Perceptron (MLP) trained on the count-based matrix

| **Department** | **hidden dims** | **dropout** | **epochs** | **patience** | **lr** | **weight decay** |
| --- | --- | --- | --- | --- | --- | --- |
| All hospital department | 512-256-128 | 0.3 | 200 | 30 | 1.00E-04 | 1.00E-04 |
| Surgery | 128-64 | 0.3 | 200 | 30 | 1.00E-04 | 1.00E-04 |
| Oncology | 256-128 | 0.3 | 200 | 30 | 3.00E-03 | 1.00E-02 |
| Rehabilitation | 512-256-128 | 0.3 | 200 | 30 | 1.00E-03 | 1.00E-03 |
| Generic | 256-128 | 0.3 | 200 | 30 | 1.00E-04 | 1.00E-04 |

# Supplementary Table S5: Best Hyperparameters for the MultiLayer Perceptron (MLP) trained on the CLMBR-T-Base embeddings

# 2 Supplementary Figures

**Supplementary Figure 1**. Mapping workflow from the Maugeri OMOP database to the foundation model representation. Overview of the process used to harmonize and map clinical data from the Italian Maugeri OMOP database to the terminology and input requirements of the foundation model. For each OMOP concept_code in the Maugeri Database, if the code is not available in the CKMBR-T-Base vocabulary, alternatives are searched based on different concept_relationship, with a maximum of three iterative steps.

**Supplementary Figure 1**. Length of stay distribution for all hospital admissions

**Supplementary Figure 2**. Length of stay distribution for hospital admissions in the surgery department

**Supplementary Figure 3**. Length of stay distribution for hospital admissions in the oncology department

**Supplementary Figure 4**. Length of stay distribution for hospital admissions in the rehabilitation department

**Supplementary Figure 5**. Length of stay distribution for hospital admissions in the all departments excluding surgery, oncology and rehabilitation

b)

a)

d)

c)

**Supplementary Figure 6**. Errors (days) for a) count-based GB, b) embedding based GB, c) count-based MLP, and d) embedding-based MLP
